# Breakthrough SARS-CoV-2 Infections, Hospitalizations, and Mortality in Vaccinated Patients With Cancer in the US Between December 2020 and November 2021

**DOI:** 10.1001/jamaoncol.2022.1096

**Published:** 2022-04-08

**Authors:** William Wang, David C. Kaelber, Rong Xu, Nathan A. Berger

**Affiliations:** 1 Center for Artificial Intelligence in Drug Discovery, School of Medicine, Case Western Reserve University, Cleveland, Ohio; 2 Center for Science, Health, and Society, School of Medicine, Case Western Reserve University, Cleveland, Ohio; 3 Departments of Internal Medicine, Pediatrics, and Population and Quantitative Health Sciences, Center for Clinical Informatics Research and Education, The MetroHealth System, Cleveland, Ohio; 4 Case Comprehensive Cancer Center, School of Medicine, Case Western Reserve University, Cleveland, Ohio

## Abstract

**Importance:**

Limited data have been presented to examine breakthrough SARS-CoV-2 infections, hospitalizations, and mortality in vaccinated patients with cancer in the US.

**Objectives:**

To examine the risk of breakthrough SARS-CoV-2 infection, hospitalizations, and mortality in vaccinated patients with cancer between December 2020 and November 2021.

**Design, Setting, and Participants:**

Retrospective cohort study of electronic health records (EHRs) of vaccinated patients from a multicenter and nationwide database in the US during the period of December 2020 through November 2021. The study population comprised patients who had documented evidence of vaccination (2 doses of Moderna or Pfizer-BioNTech or single dose of Janssen/Johnson & Johnson vaccines) in their EHRs from December 2020 to November 2021 and had no SARS-CoV-2 infection prior to vaccination.

**Exposures:**

The 12 most common cancers combined and separately; recent vs no recent encounter for cancer; and breakthrough SARS-CoV-2 infection.

**Main Outcomes and Measures:**

Time trends of incidence proportions of breakthrough SARS-CoV-2 infections from December 2020 to November 2021 in vaccinated patients with all cancer; cumulative risks of breakthrough infections in vaccinated patients for all cancer and 12 common cancer types; hazard ratios (HRs) and 95% CIs of breakthrough infections between propensity score–matched patients with vs without cancer and between propensity score–matched patients with cancer who had a recent medical encounter for cancer vs those who did not; overall risks, HRs, and 95% CIs of hospitalizations and mortality in patients with cancer who had breakthrough infections vs those who did not.

**Results:**

Among 45 253 vaccinated patients with cancer (mean [SD] age, 68.7 [12.4] years), 53.5% were female, 3.8% were Asian individuals, 15.4% were Black individuals, 4.9% were Hispanic individuals, and 74.1% were White individuals. Breakthrough SARS-CoV-2 infections in patients with cancer increased from December 2020 to November 2021 and reached 52.1 new cases per 1000 persons in November 2021. The cumulative risk of breakthrough infections in patients with all cancer was 13.6%, with highest risk for pancreatic (24.7%), liver (22.8%), lung (20.4%), and colorectal (17.5%) cancers, and lowest risk for thyroid (10.3%), endometrial (11.9%), and breast (11.9%) cancers, vs 4.9% in the noncancer population (*P* < .001). Patients with cancer had significantly increased risk for breakthrough infections vs patients without cancer (HR, 1.24; 95% CI, 1.19-1.29), with greatest risk for liver (HR, 1.78; 95% CI, 1.38-2.29), lung (HR, 1.73; 95% CI, 1.50-1.99), pancreatic (HR, 1.64; 95% CI, 1.24-2.18), and colorectal (HR, 1.53; 95% CI, 1.32-1.77) cancers and lowest risk for thyroid (HR, 1.07; 95% CI, 0.88-1.30) and skin (HR, 1.17; 95% CI, 0.99-1.38) cancers. Patients who had medical encounters for cancer within the past year had higher risk for breakthrough infections than those who did not (HR, 1.24; 95% CI, 1.18-1.31). Among patients with cancer, the overall risk for hospitalizations and mortality was 31.6% and 3.9%, respectively, in patients with breakthrough infections, vs 6.7% and 1.3% in those without breakthrough infections (HR for hospitalization: 13.48; 95% CI, 11.42-15.91; HR for mortality: 6.76; 95% CI, 4.97-9.20).

**Conclusions and Relevance:**

This cohort study showed significantly increased risks for breakthrough infection in vaccinated patients with cancer, especially those undergoing active cancer care, with marked heterogeneity among specific cancer types. Breakthrough infections in patients with cancer were associated with significant and substantial risks for hospitalizations and mortality.

## Key Points

**Question:**

What are the time trends, risks, and outcomes of breakthrough SARS-CoV-2 infections in vaccinated patients with cancer in the US between December 2020 and November 2021?

**Findings:**

In this cohort study of a nationwide database of electronic health records of 636 465 vaccinated patients, the 45 253 vaccinated patients with cancer had significantly higher risk for breakthrough SARS-CoV-2 infections than propensity score–matched patients without cancer, with marked heterogeneity among 12 common cancers, including solid tumors and hematologic cancers, most common in patients with active cancer within the past year. Breakthrough infections in patients with cancer were associated with significant and substantial risks for hospitalizations and mortality.

**Meaning:**

With the emergence of SARS-CoV-2 virus variants and the waning immunity of vaccines, the findings raise the consideration for the development and implementation of enhanced mitigation strategies in vaccinated patients with specific cancers, especially those undergoing active cancer care.

## Teaser

This cohort study examines the risk of breakthrough SARS-CoV-2 infection, hospitalizations, and mortality due to COVID-19 in vaccinated patients with cancer compared with vaccinated patients without cancer between December 2020 and November 2021.

## Introduction

Data from early in the pandemic, when vaccines were not available, showed that patients with cancer were at increased risk for COVID-19 and severe outcomes.^1,2,3,4^ COVID-19 vaccines are effective against infections and outcomes in real-world populations,^5,6^ including those with cancer.^7^ However, breakthrough infections have been recorded.^8,9,10,11,12^ Studies have shown that patients with cancers or active cancer treatments had poor antibody response to COVID-19 messenger RNA vaccines compared with healthy controls,^13,14,15,16^ with antibody titers declining significantly after 36 days.^17^ In contrast, another study showed that patients with cancer including those on active treatments developed an adequate antibody response to COVID-19 vaccines.^18^ However, there have been limited nationwide real-world data presented to systematically examine breakthrough SARS-CoV-2 infections, hospitalizations, and mortality in vaccinated patients with cancer and how the risks for breakthrough infections vary among different cancer types. In this study of a large, geographically diverse real-time database of patient electronic health records (EHRs) in the US, we characterize and quantify breakthrough infections, hospitalizations, and mortality in vaccinated patients for both all cancers and the 12 most common cancers in the US.

## Methods

### Database Description

We used the TriNetX Analytics network platform, which contains deidentified data of 90 million unique patients from 66 health care organizations, mostly large academic medical institutions with both inpatient and outpatient facilities at multiple locations, across 50 states in the US,^19^ covering diverse geographic locations, age groups, racial and ethnic groups, income levels, and insurance types. End users can use the TriNetX web portal to send queries to the Advanced Analytics Platform to perform cohort selection, propensity score matching, and time trend analysis to compare outcomes between cohorts. Although the data are deidentified, the built-in statistical functions within TriNetX Analytics Platform can perform statistical analyses on the patient-level data. TriNetX only reports on population-level data without including protected health information identifiers. The MetroHealth System, Cleveland, Ohio, institutional review board has determined that any research using TriNetX is not human participants research and therefore is exempt from institutional review board review (more details in eMethods in the Supplement). We have previously used the TriNetX database to study SARS-CoV-2 infections in the US.^11,12,20^ This study followed the Strengthening the Reporting of Observational Studies in Epidemiology (STROBE) reporting guidelines.^21^

### Study Population

The study population comprised 45 253 vaccinated patients with a diagnosis of at least 1 of the 12 common cancer types that are diagnosed with the greatest frequency in the US—bladder, breast, colorectal, endometrial, hematologic, kidney, liver, lung, pancreatic, prostate, skin, and thyroid^22^—and 590 914 vaccinated patients without cancer. The population fulfilled the following inclusion criteria: had documented evidence of vaccination in the EHRs (received 2 doses of COVID-19 messenger RNA vaccine, or single dose of the COVID-19 viral vector vaccine) between December 2020 and November 2021 and did not contract SARS-CoV-2 infection prior to vaccination. Race and ethnicity were defined by the TriNetX EHR database and included in the study because they are known to be associated with both risk and associated outcomes of SARS-CoV-2 infections.

The status of the 12 cancer types was determined based on *International Statistical Classification of Diseases and Related Health Problems, Tenth Revision (ICD-10)* codes (details in eTable 1 in the Supplement). The status of all cancer was based on patients with at least 1 of the aforementioned 12 cancer types. The status of noncancer was based on patients without the *ICD-10* diagnosis code of “neoplasm” (C00-D49). The status of breakthrough SARS-CoV-2 infection was based on laboratory-test confirmed presence of “SARS coronavirus 2 and related RNA” (TNX:LAB:9088) or the *ICD-10* diagnosis code of “COVID-19” (U07.1). The status of COVID-19 vaccination was based on *Current Procedural Terminology *codes (details in eTable 2 in the Supplement). The status of hospitalization was based on *Current Procedural Terminology *code “hospital inpatient services” (013659) and death on the vital status code “deceased” that TriNetX regularly imports from the Social Security Death Index.

### Statistical Analysis

The list of covariates, their standardized names, codes, and data types that are used in the TriNetX database are described in eTable 2 in the Supplement. These covariates included demographics (age, sex, race and ethnicity) that were based on the underlying clinical EHR systems of the contributing health care systems; adverse socioeconomic determinants of health (SDOHs) that were based on *ICD-10* codes; comorbidities that are related to COVID-19 risks or outcomes^23,24^; behavioral factors (tobacco smoking, alcohol abuse); vaccine types; and cancer treatment types.

1. We examined monthly incidence proportions of breakthrough SARS-CoV-2 infections (new cases per 1000 persons) between December 2020 and November 2021 in vaccinated patients with all cancer and in patients without cancer. Specific cancer types were not examined owing to small sample sizes.
2. The cumulative risks of breakthrough SARS-CoV-2 infections between December 2020 and November 2021 were examined and compared between White and Black patients, and between Hispanic and non-Hispanic patients, in vaccinated patients for all cancer, 12 specific cancers, and noncancer.
3. We investigated if cancer as a disease entity was associated with increased risks for breakthrough SARS-CoV-2 infections in vaccinated populations by comparing patients with cancer vs patients without cancer, with patients being propensity score matched (1:1 using a nearest neighbor greedy matching^25^) for demographics, SDOHs, comorbidities, and vaccine types (eTable 2 in the Supplement). Separate analyses were performed for all cancer and for each of the 12 cancers. Breakthrough infections in matched cohorts were followed starting 14 days after vaccination until November 30, 2021. Kaplan-Meier analysis was used to estimate the probability of breakthrough infections. The Cox proportional hazards model was used to compare the 2 matched cohorts. The proportional hazard assumption was tested using the generalized Schoenfeld approach. Hazard ratios (HRs) and 95% CIs were used to describe the relative hazard of breakthrough infections based on comparison of time-to-event rates.
4. We examined whether patients who had recent medical encounters for cancer had different risk profiles for breakthrough infections from patients who did not have recent medical encounters for cancer. Patients with cancer were stratified into 2 dichotomous cohorts: (1) cohort 1 had medical encounters for cancer within the past year, and (2) cohort 2 had no medical encounters for cancer within the past year (November 2020-November 2021). Two cohorts were propensity score matched for covariates listed in eTable 2 in the Supplement. The HRs and 95% CIs for breakthrough infections in matched cohorts were calculated as described above. Separate analysis was done for all cancer and for each of the 12 cancer types.
5. The overall risks for hospitalizations and mortality in patients who had breakthrough infections (breakthrough cohort) were examined and compared with patients who had no breakthrough infections (nonbreakthrough cohort) for all cancer as well as noncancer populations. Two cohorts were propensity score matched for the covariates listed in eTable 2 in the Supplement and 12 cancer types (for cancer cohorts). Individual cancer type was not examined owing to small sample sizes of patients with severe outcomes. Outcomes were followed starting on the day of infection for the breakthrough cohorts and 14 days after vaccination for the nonbreakthrough cohorts. The HRs and 95% CIs for hospitalizations and mortality in matched cohorts were calculated as described above.

All statistical tests were conducted within the TriNetX Advanced Analytics Platform on December 17, 2021, at significance set at *P* < .05 (2-sided). The TriNetX platform calculates HRs and associated CIs using R’s Survival package, version 3.2-3, with the proportional hazard assumption tested using the generalized Schoenfeld approach. Details of the TriNetX database, study population, and statistical methods are in eMethods in the Supplement.

## Results

### Patient Characteristics

The study population of vaccinated population comprised 45 253 vaccinated patients with at least 1 of 12 cancer types (“all cancer”) and 591 212 vaccinated patients without cancer. Among 45 253 vaccinated patients with cancer (mean [SD] age, 68.7 [12.4] years), 53.5% were female, 3.8% were Asian individuals, 15.4% were Black individuals, 4.9% were Hispanic individuals, and 74.1% were White individuals. Among 591 212 patients without cancer (mean [SD] age, 51.1 [20.9] years), 55.1% were female, 8.3% were Asian individuals, 14.2% were Black individuals, 12.2% were Hispanic individuals, and 62.7% were White individuals. The characteristics of the study population are shown in Table 1 and eTable 3 in the Supplement. Compared with patients without cancer, patients with cancer were older and had more comorbidities and SDOHs.

**Table 1.** Characteristics of 45 253 Vaccinated Patients With Cancer and 591 212 Vaccinated Patients Without Cancer^a^

Characteristic	Patients, %							
	Cancer type						All cancer	Not cancer
	Breast	Prostate	Hematologic	Colorectal	Skin	Lung		
Total No. of patients	13 032	11 421	6962	3094	2926	2849	45 253	591 212
Age, mean (SD), y	68.3 (11.7)	72.4 (8.85)	65.9 (15.6)	70.1 (12.3)	68.1 (13.5)	71.4 (10.7)	68.7 (12.4)	51.1 (20.9)
Sex								
Female	99.0	0.2	48.8	50.8	48.6	57.7	53.5	55.1
Male	1.0	99.8	51.2	49.2	51.4	42.3	46.5	44.9
Ethnicity								
Hispanic	4.9	4.2	5.6	5.6	1.8	2.7	4.9	12.2
Non-Hispanic	82.5	84.5	81.5	79.3	84.6	78.8	81.8	73.9
Unknown	12.7	11.3	12.8	15.2	13.6	18.5	13.3	13.8
Race								
Asian	4.5	3.0	3.4	3.7	0.7	3.9	3.8	8.3
Black	15.6	18.4	14.9	16.8	1.6	17.1	15.4	14.2
White	73.3	72.3	73.8	73.1	92.5	74.1	74.1	62.7
Unknown	6.2	5.9	7.5	5.9	5.1	4.7	6.3	13.8
Medical encounter for cancer within past year	58.0	61.3	60.6	46.6	38.3	62.8	58.5	NA

^a^ Shown were the 6 most common cancer types in the database. Race and ethnicity were defined by the TriNetX electronic health record database and included in the study because they are known to be associated with both risk and associated outcomes of SARS-CoV-2 infections.

### Monthly Incidence Proportions of Breakthrough SARS-CoV-2 Infections in Vaccinated Patients

**Figure 1.**
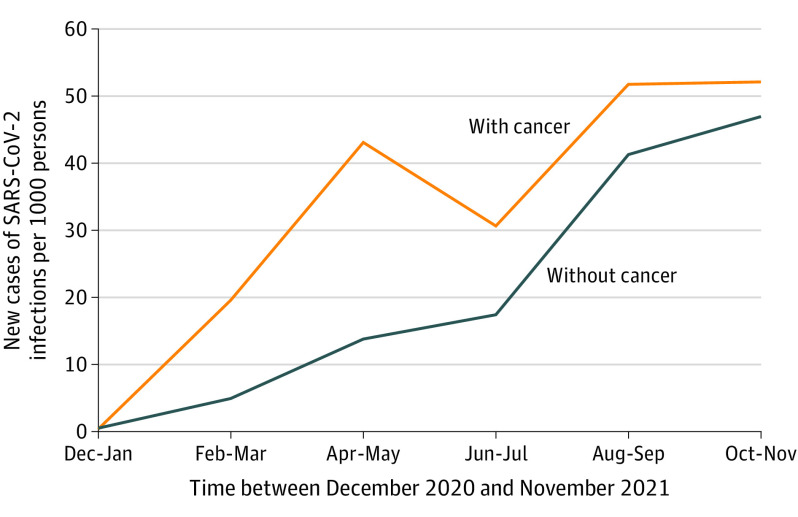
Time Trend of Incidence Proportions (New Cases per 1000 Persons) of Breakthrough SARS-CoV-2 Infections From December 2020 to November 2021 in Vaccinated Patients With All Cancer and Patients Without Cancer

The monthly incidence proportions of breakthrough SARS-CoV-2 infections (measured by new cases per 1000 persons) in vaccinated patients with cancer and in vaccinated patients without cancer steadily increased from December 2020 to November 2021 (Figure 1). The proportions were significantly higher in patients with cancer than in patients without cancer: 19.6 vs 4.9 in February to March (*P* < .001), 43.1 vs 13.8 in April to May (*P* < .001), 30.6 vs 17.4 in June to July (*P* < .001), 51.7 vs 41.3 in August to September (*P* < .001), and 52.1 vs 46.9 in October to November (*P* < .001) (Figure 1).

### Cumulative Risks of Breakthrough SARS-CoV-2 Infections in Vaccinated Patients

The cumulative risk of breakthrough infections in vaccinated patients with all cancer during the study period of December 2020 to November 2021 was 13.6%. Among the 12 cancer types, patients with pancreatic cancer had the highest risk (24.7%), followed by patients with liver cancer (22.8%), lung cancer (20.4%), and colorectal cancer (17.5%), with lowest risk for thyroid cancer (10.3%), endometrial cancer (11.9%), and breast cancer (11.9%), all significantly higher than the 4.9% in patients without cancer (*P* < .001) (Table 2). The overall risks did not differ based on race or ethnicity for 12 specific cancer types. Racial and ethnic disparity was observed in patients with all cancer and in patients without cancer; however, these 2 populations were heterogeneous in cancer types and other diseases.

**Table 2.** Cumulative Risks of Breakthrough SARS-CoV-2 Infections in Patients With Cancer

Cohort	Patients in cohort	Risk, %				
		Overall	Black patients	White patients	Hispanic patients	Non-Hispanic patients
All cancer	45 253	13.6	13.8	13.9	16.0	13.9^a^
Cancer type						
Breast	13 032	11.9	11.4	12.5	12.8	12.2
Prostate	11 422	12.8	12.6	13.5	15.1	12.7
Hematologic	6959	14.9	16.2	14.3	16.5	14.6
Colorectal	3094	17.5	17.2	16.9	21.1	17.4
Skin	2926	12.5	NA^b^	12.1	NA^b^	13.0
Lung	2849	20.4	19.7	20.5	22.4	20.6
Thyroid	2329	10.3	9.7	10.0	13.4	9.5
Bladder	2253	17.4	18.3	16.1	24.7	16.2
Kidney	2075	16.0	19.0	15.6	17.1	15.1
Endometrial	1684	11.9	12.6	11.3	14.1	11.9
Liver	873	22.8	21.2	22.6	32.1	23.4
Pancreatic	619	24.7	25.5	24.5	25.2	NA^b^
Not cancer	591 212	4.9	5.3	5.3	4.3	5.2^a^

Abbreviation: NA, not available.

^a^ Denotes that the difference between Black patients and White patients or between Hispanic patients and non-Hispanic patients was significant (*P* < .05).

^b^ TriNetX does not report actual patient counts less than 10 for security reasons.

### Comparison of Breakthrough SARS-CoV-2 Infections in Propensity Score–Matched Patients: Cancer vs Noncancer

**Figure 2.**
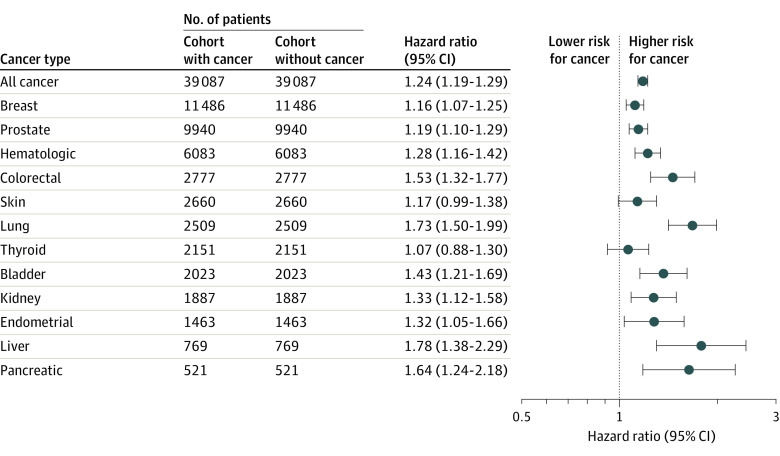
Hazard Ratios for Breakthrough SARS-CoV-2 Infections in Vaccinated Patients With Cancer (Cancer Cohort) Compared With Propensity Score–Matched Patients Without Cancer (Noncancer Cohort) Breakthrough SARS-CoV-2 infections were followed in both cohorts starting 14 days after full vaccination up to November 30, 2021. Two cohorts were matched for demographics (age, sex, race and ethnicity), socioeconomic determinants of health, comorbidities, and vaccine types.

Vaccinated patients with all cancer had significantly higher risk for breakthrough infections than patients without cancer after matching for demographics, SDOHs, comorbidities, and vaccine types (HR, 1.24; 95% CI, 1.19-1.29). Among 12 specific cancer types, the greatest risk was for liver cancer (HR, 1.78; 95% CI, 1.38-2.29), followed by lung cancer (HR, 1.73; 95% CI, 1.50-1.99), pancreatic cancer (HR, 1.64; 95% CI, 1.24-2.18), and colorectal cancer (HR, 1.53; 95% CI, 1.32-1.77), and the lowest risk was for thyroid cancer (HR, 1.07; 95% CI, 0.88-1.30), skin cancer (HR, 1.17; 95% CI, 0.99-1.38), breast cancer (HR, 1.16; 95% CI, 1.07-1.25), and prostate cancer (HR, 1.19; 95% CI, 1.10-1.29) (Figure 2). These findings suggest that cancer as a disease entity is a risk factor for breakthrough infections in vaccinated patients, with heterogeneous effects among 12 cancer types.

### Comparison of Breakthrough SARS-CoV-2 Infections in Propensity Score–Matched Patients With Cancer: Recent vs No Recent Medical Encounters for Cancer

Among vaccinated patients with all cancer, individuals who had medical encounters for their cancer within the past year had higher risk for breakthrough infections than those who did not, after matching for cancer types, demographics, SDOHs, comorbidities, vaccine types, and cancer treatments (HR, 1.24; 95% CI, 1.18-1.31). Increased risks were observed for breast cancer (HR, 1.40; 95% CI, 1.26-1.54), hematologic cancers (HR, 1.39; 95% CI, 1.23-1.58), colorectal cancer (HR, 1.76; 95% CI, 1.48-2.08), bladder cancer (HR, 1.35; 95% CI, 1.12-1.64), and pancreatic cancer (HR, 1.75; 95% CI, 1.06-2.88), but not for other cancer types (eFigure in the Supplement).

### Comparison of Hospitalizations and Mortality in Vaccinated Patients: Breakthrough vs No Breakthrough Infections

**Figure 3.**
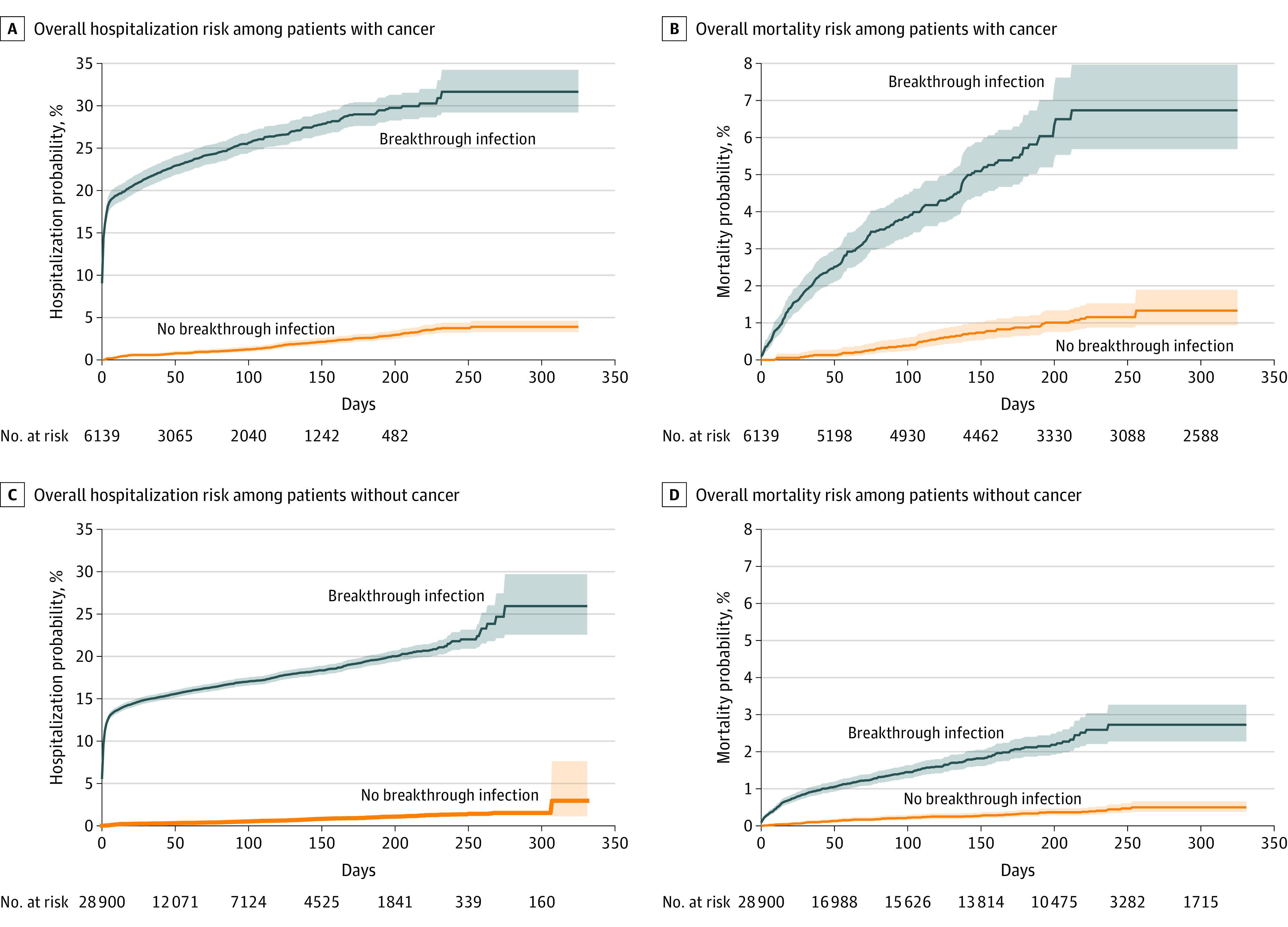
Overall Risks of Hospitalization and Mortality in Patients With Breakthrough Infection vs Patients Without Breakthrough Infections Kaplan-Meier curves for overall risks for hospitalizations in patients with cancer who had breakthrough infections and in matched patients with cancer who had no breakthrough infection (the numbers at risk at 205, 217, and 232 days were 349, 217, and 91, respectively) (A); overall mortality in patients with cancer who had breakthrough infections and in matched patients with cancer who had no breakthrough infections (the number at risk at 325 days was 2588) (B); overall risks for hospitalizations in patients without cancer who had breakthrough infections and in matched patients with cancer who had no breakthrough infections (C); and overall mortality in patients without cancer who had breakthrough infections and in matched patients without cancer who had no breakthrough infections (D). Shaded areas represent 95% CIs.

Among patients with all cancer, the overall hospitalization risk was 31.6% in patients with breakthrough infections, higher than 3.9% in propensity score–matched patients without breakthrough infections (HR, 13.48; 95% CI, 11.42-15.91) (Figure 3A). The overall mortality risk was 6.7% in patients with cancer who had breakthrough infections, higher than 1.3% in matched patients with cancer who had no breakthrough infections (HR, 6.76; 95% CI, 4.97-9.20) (Figure 3B).

Among patients without cancer, the overall hospitalization risk was 25.9% in patients with breakthrough infections, higher than the risk of 3.0% in matched patients without breakthrough infections (HR, 27.70; 95% CI, 24.09-31.85) (Figure 3C). The overall mortality risk was 2.7% in patients without cancer who had breakthrough infections, higher than 0.5% in matched patients without cancer who had no breakthrough infections (HR, 6.77; 95% CI, 5.19-8.83) (Figure 3D).

## Discussion

This study shows that the incidence proportions of breakthrough SARS-CoV-2 infections in vaccinated in patients with all cancer as well as in patients without cancer continuously increased from December 2020 to November 2021. This increasing time trend may reflect waning immunity of vaccines, the emergence of different virus variants, and varied measures taken by individuals and communities over time during the pandemic, among other factors. The incidence proportion of breakthrough infections was significantly higher in patients with cancer, indicating that vaccines are less protective against SARS-CoV-2 infection in patients with cancer and/or that patients with cancer are more susceptible to SARS-CoV-2 infection. In either case, patients with cancer are at increased risk for breakthrough SARS-CoV-2 infection.

To determine if cancer as a disease entity is a risk factor for breakthrough infections, we compared the risks for breakthrough infections between patients with cancer and those without cancer after propensity score matching for demographic characteristics, SDOHs, comorbidities, and vaccine types. We show that of 12 cancers studied, all except thyroid and skin cancer were associated with significantly increased risks for breakthrough infections. The marked heterogeneity in breakthrough infections for specific cancer types further indicates that cancer is a risk factor for breakthrough infections in the vaccinated population. Patients with hematologic cancers have impaired antibody response to vaccines owing to their compromised immune functions,^14,15,16^ lower than that in patients with solid tumors.^17^ Interestingly, findings from this study showed that the overall risk for breakthrough infection among patients with hematologic cancers was lower than that for patients with colorectal, lung, bladder, kidney, liver, and pancreatic cancer. These results suggest that the levels of impaired antibody response to COVID-19 vaccines in patients with cancer may not directly correlate with breakthrough infections. During the early stage of the pandemic when vaccines were not available, we reported significant racial and ethnic disparities in COVID-19 risk in individuals with cancer.^1,2^ In this study, no significant racial and ethnic difference in risks for breakthrough infections was observed in vaccinated patients with cancer for all 12 cancer types, suggesting that vaccination may have narrowed or eliminated the racial and ethnic disparities in SARS-CoV-2 infections in patients with cancer for the population in this study.

Results of this study show that vaccinated patients with cancer who had medical encounters for their cancer in the past year had higher risk for breakthrough infections than those who did not, after matching for cancer types, demographics, SDOHs, comorbidities, vaccine types, and cancer treatments (HR, 1.24; 95% CI, 1.18-1.31), with similar trends observed for breast, hematologic, colorectal, bladder, and pancreatic cancer. Patients with a diagnosis of cancer who had medical encounters for cancer in the past year may represent patients with active cancer. On the other hand, patients with a diagnosis of cancer who did not have medical encounters for cancer in the past year may represent patients with less active cancer or patients who had a history of cancer but now are cancer free. Nonetheless, these results further indicate that active cancer itself and/or active cancer treatments may have played important roles. Future research is necessary to further disentangle the effects of active cancer itself from those from active cancer treatments.

Findings from this study showed that breakthrough SARS-CoV-2 infection resulted in substantial risks for severe outcomes, including mortality, in vaccinated patients with cancer and those without cancer. Among vaccinated patients with cancer, the overall mortality risk was 6.7% in patients with breakthrough infections, compared with 1.3% in matched patients without breakthrough infections (HR, 6.76; 95% CI, 4.97-9.20). While the causes of death were unknown, this finding suggests that the significant extra mortality was likely attributable to breakthrough infections. Similar extra mortality that was likely attributable to breakthrough infections was observed in patients without cancer. Among vaccinated patients without cancer, the overall mortality risk was 2.7% in patients with breakthrough infections, compared with 0.5% in matched patients without breakthrough infections (HR, 6.77; 95% CI, 5.19-8.83). As expected, patients with cancer had higher mortality than patients without cancer, for both patients with breakthrough infection (6.7% for cancer vs 2.7% for noncancer) and patients without breakthrough infection (1.3% for cancer vs 0.5% for noncancer).

### Limitations

This study has several limitations. First, the observational, retrospective nature of this study could introduce biases related to case selection, reporting, testing and follow-up. However, because we compared different cohort populations all from within the TriNetX data set, these issues should not substantially affect the relative risk analyses. Second, time-series antibody level data in vaccinated patients are not available in patient EHRs; therefore, we could not correlate antibody data with breakthrough infections, hospitalizations, and death to advance our understanding of the immune correlates of protection in patients with cancer. Third, patients in the TriNetX EHR database represent people who had medical encounters with the 66 contributing health care systems, most of which are large academic medical institutions. Therefore, the generalizability of our findings needs to be validated in other populations and in other national data resources, such as the National COVID Cohort Collaborative (N3C), the National Patient-Centered Clinical Research Network (PCORnet), Consortium for Clinical Characterization of COVID-19 by EHR (4CE), the national Accrual to Clinical Trials (ACT) Network, and Optum EHR data. Fourth, we used relevant *ICD-10* diagnosis codes as an approximation for SDOHs. The prevalence of these codes was significantly higher in patients with cancer than those without cancer, and there is marked difference for different cancer types. While the data are consistent with our current understanding that modifiable environmental and SDOHs are substantial contributing risk factors for cancer in general and that the degrees of their effects vary for different cancer types,^26^ the overall prevalence of these approximate SDOH codes as documented in patients’ EHRs is low. However, these issues should not substantially affect the relative risk analyses. Fifth, COVID-19 vaccination rates in the TriNetX database may underrepresent true COVID-19 vaccination rates because many patients are receiving their COVID-19 vaccines outsides of health care systems, and not all health care systems contributing data to TriNetX are submitting state immunization registry codes to TriNetX. However, this issue of underreporting of vaccination should not affect our results because this study focused on vaccinated patients. Finally, we showed that patients who had recent medical encounters for cancer had higher risks for breakthrough infections than patients who did not, by splitting patients with cancer into 2 dichotomous cohorts owing to small sample sizes. However, how timing of cancer treatments, cancer diagnosis, and disease characteristics and other cancer-related features affect the risks of breakthrough infections and severe outcomes in vaccinated patients with cancer warrant further investigation.

## Conclusions

Findings from this cohort study showed significantly increased risks for breakthrough SARS-CoV-2 infection in vaccinated patients with cancer, with marked heterogeneity among specific cancer types. Breakthrough infections were associated with substantial hospitalizations and mortality in vaccinated patients with cancer. These results emphasize the need for patients with cancer to maintain mitigation practice, especially with the emergence of different virus variants and the waning immunity of vaccines. Future studies are warranted to continue to evaluate breakthrough infections and associate severe outcomes in vaccinated patients with cancer and to understand the biological mechanisms underlying the marked heterogeneity in breakthrough infections among different cancer types.

## Notes

Supplement.
eMethods.
**eTable 1.** Cancer types and their standardized names, ICD-10 codes and data types that are used in the TriNetX database **eTable 2.** Covariates and their standardized names, codes and data types that are used in the TriNetX database **eTable 3.** Characteristics of 45,253 vaccinated patients with cancer and 591,212 vaccinated patients without cancer **eFigure.** Comparison of breakthrough infections in vaccinated patients with cancer (recent vs no recent medical encounters for cancer diagnosis)
