# Supplementary material for: Breakthrough SARS-CoV-2 Infections, Hospitalizations, and Mortality in Vaccinated Patients With Cancer in the US Between December 2020 and November 2021
Source: JAMA Oncol. 2022 Apr 8;8(7):1027–34. doi: 10.1001/jamaoncol.2022.1096 (PMC9305383; doi:10.1001/jamaoncol.2022.1096)
Supplement: Supplement. — eMethods. eTable 1. Cancer types and their standardized names, ICD-10 codes and data types that are used in the TriNetX database eTable 2. Covariates and their standardized names, codes and data types that are used in the TriNetX database eTable 3. Characteristics of 45,253 vaccinated patients with cancer and 591,212 vaccinated patients without cancer eFigure. Comparison of breakthrough infections in vaccinated patients with cancer (recent vs no recent medical encounters for cancer diagnosis) [file jamaoncol-e221096-s001.pdf]

Supplemental Online Content

Wang W, Kaelber DC, Xu R, Berger NA. Breakthrough SARS-CoV-2 infections, hospitalizations, and mortality in vaccinated patients with cancer in the US between December 2020 and November 2021. *JAMA Oncol*. Published online April 8, 2022. doi:10.1001/jamaoncol.2022.1096

eMethods.

eTable 1. Cancer types and their standardized names, ICD-10 codes and data types that are used in the TriNetX database

eTable 2. Covariates and their standardized names, codes and data types that are used in the TriNetX database

eTable 3. Characteristics of 45,253 vaccinated patients with cancer and 591,212 vaccinated patients without cancer

eFigure. Comparison of breakthrough infections in vaccinated patients with cancer (recent vs no recent medical encounters for cancer diagnosis)

This supplemental material has been provided by the authors to give readers additional information about their work.

© 2022 Wang W et al. *JAMA Oncology*.

eMethods

Description of TriNetX database

The data used in this study was collected on December 17, 2021 from the TriNetX COVID-19 Research Network, which provided access to electronic medical records (diagnoses, procedures, medications, laboratory values, genomic information) from approximately 90 million patients from 66 healthcare organizations. TriNetX, LLC is compliant with the Health Insurance Portability and Accountability Act (HIPAA), the US federal law which protects the privacy and security of healthcare data, and any additional data privacy regulations applicable to the contributing HCO. TriNetX is certified to the ISO 27001:2013 standard and maintains an Information Security Management System (ISMS) to ensure the protection of the healthcare data it has access to and to meet the requirements of the HIPAA Security Rule. Any data displayed on the TriNetX Platform in aggregate form, or any patient level data provided in a data set generated by the TriNetX Platform, only contains de-identified data as per the de-identification standard defined in Section §164.514(a) of the HIPAA Privacy Rule. The process by which the data is de-identified is attested to through a formal determination by a qualified expert as defined in Section §164.514(b)(1) of the HIPAA Privacy Rule. Because this study used only de-identified patient records and did not involve the collection, use, or transmittal of individually identifiable data, this study was exempted from Institutional Review Board approval. We reviewed this assessment with MetroHealth System, Cleveland, Ohio, IRB staff and they have agreed that any research using TriNetX, based on the details provided above is not Human Subject Research and therefore exempt from IRB review.

TriNetX is a platform that de-identifies and aggregates electronic health record (EHR) data from 66 contributing healthcare systems, most of which are large academic medical institutions with both inpatient and outpatient facilities at multiple locations, across 50 states in the US. TriNetX Analytics provides web-based and secure access to patient EHR data from hospitals, primary care, and specialty treatment providers, covering diverse geographic locations, age groups, racial and ethnic groups, income levels and insurance types including various commercial insurances, governmental insurance (Medicare and Medicaid), self-pay/uninsured, worker compensation insurance, military/VA insurance among others. The database contains EHR data and there is no linking of claims data. Any data that may appear to be claims data is coming from the EHR itself, which has one of it purposes to generate a claim. Although the sample is not a “random sample” of the population of people living in the US, it is a convenient sample, of over 1/5th of the people living in the US, all of who obtain healthcare at a facility with a contributing EHR, more skewed to patients being seen within academic medical centers.

The contributing EHR systems used United Medical Language System (UMLS) biomedical ontologies for coding. TriNetX maps the data to a standard and controlled set of clinical terminologies, for example, mapping disease terms from SNOMED-CT to ICD-10, drug terms from NDCs to RxNorm. The data is then transformed into a proprietary data schema. This transformation process includes an extensive data quality assessment that includes data cleaning.

© 2022 Wang W et al. *JAMA Oncology.*

Statistical analysis

Monthly incidence proportion of breakthrough COVID-19 infection between 12/2020-11/2021 were examined in among 45,253 vaccinated patients with at least one of the 12 cancer types ("all cancer"). In this study, incidence is a rate of breakthrough COVID-19 infection cases in the population under analysis. For each month, the incidence proportion denominator includes all and only those patients in the cohort under analysis, whose fact record overlaps the time window by at least one day and who did not have COVID-19 any time before the time window. The incidence proportion numerator includes all and only those patients who are in the denominator and who had COVID-19 within the time window.

We tested whether the risk for breakthrough infections differed between vaccinated patients with vs. without cancer, and between vaccinated patients with cancer who had vs. who did not have medical encounter for cancer within the past year. Patient cohorts were first propensity-score matched (1:1 using a nearest neighbor greedy matching with a caliper of 0.25 times the standard deviation) for covariates in eTable2. Risks of breakthrough COVID-19 infection starting 14 days after vaccination until November 30, 2021 were compared between matched cohorts with hazard ratios and 95% confidence intervals. Kaplan-Meier analysis was used to estimate the probability of breakthrough COVID-19 infections. Cox's proportional hazards model was used to compare the two matched cohorts. The proportional hazard assumption was tested using the generalized Schoenfeld approach. We also tested whether risks for hospitalization and mortality differed between patients with vs. without breakthrough infections. Similar analysis was performed to compare hospitalization and mortality between patients with vs. without breakthrough infections with patients matched for covariates in eTable2, cancer types and cancer treatment types. Outcomes were followed starting on the day of infection for the breakthrough cohorts and 14 days after vaccination for the non-breakthrough cohorts. The TriNetX Platform calculates the hazard ratios and associated confidence intervals using R's Survival package v3.2-3. For generating hazard ratios, TriNetX sets robust=FALSE using the R survival package, which is a limitation of the TriNetX platform since it does not take into account potential clustering of COVID-19 cases within the healthcare organizations or specific geolocations, partly due to TriNetX's obligation to limit the visibility of data by HCO source. All Statistical tests were conducted on 12/17/2021 within the TriNetX Analytics Platform with significance set at p-value < 0.05 (two-sided).

© 2022 Wang W et al. *JAMA Oncology.*

eTable 1: Cancer types and their standardized names, ICD-10 codes and data types that are used in the TriNetX database.

Cancer type	Name, ICD-19 code	Data type
Bladder cancer	Malignant neoplasm of bladder (C67)	present/absent
Breast cancer	Malignant neoplasm of breast (C50)	present/absent
Colorectal cancer	Malignant neoplasm of colon (C18)<br>Malignant neoplasm of rectum (C20),	present/absent
Endometrial cancer	Malignant neoplasm of endometrium (C54.1)	present/absent
Hematological malignancy	Malignant neoplasms of lymphoid, hematopoietic and related tissue (C81-C96)	present/absent
Kidney cancer	Malignant neoplasm of renal pelvis (C65)<br>Malignant neoplasm of kidney, except for renal pelvis (C64)	present/absent
Liver cancer	Malignant neoplasm of liver and intrahepatic bile ducts (C22)	present/absent
Lung cancer	Malignant neoplasm of bronchus and lung (C34)	present/absent
Pancreatic cancer	Malignant neoplasm of pancreas (C25)	present/absent
Prostate cancer	Malignant neoplasm of prostate (C61)	present/absent
Skin cancer	Malignant melanoma of skin (C43)	present/absent
Thyroid cancer	Malignant neoplasm of thyroid gland (C73)	present/absent

© 2022 Wang W et al. *JAMA Oncology.*

eTable 2: Covariates and their standardized names, codes and data types that are used in the TriNetX database.

Covariate	Name, code	Data type
Age at Index	Age at Index	continuous
Female	F	present/absent
Male	M	present/absent
Asian	Asian (Demographics: 2028-9)	present/absent
Black or African American	Black or African American (Demographics: 2054-5)	present/absent
White	White (Demographics: 2106-3)	present/absent
Hispanic/Latino	Hispanic or Latino (Demographics: 2135-2)	present/absent
Not Hispanic or Latino	Not Hispanic or Latino (Demographics: 2186-5)	present/absent
Adverse socioeconomic determinants of health	Persons with potential health hazards related to socioeconomic and psychosocial circumstances (ICD-10 code: Z55-Z65)<br>Z55: Problems related to education and literacy<br>Z56: Problems related to employment and unemployment<br>Z57: Occupational exposure to risk factors (noise, radiation, dust, other air contaminants, toxic agents in agriculture, extreme temperature, etc).<br>Z58: Problems related to physical environment<br>Z59: Problems related to housing and economic circumstances<br>Z60: Problems related to social environment<br>Z62: Problems related to upbringing<br>Z63: Other problems related to primary support groups<br>Z64-Z65: Problems related to psychosocial circumstances	present/absent
Hypertension	Hypertensive diseases (ICD-10 code: I10-I16)	present/absent
Heart diseases	Ischemic heart diseases (ICD-10 code: I20-I25)	present/absent
Cerebrovascular diseases	Cerebrovascular diseases (ICD-10 code: I60-I69)	present/absent
Obesity/overweight	Overweight and obesity (ICD-10 code: E66)	present/absent
Type 2 diabetes	Type 2 diabetes mellitus (ICD-10 code: E11)	present/absent
Chronic respiratory diseases	Chronic lower respiratory diseases (ICD-10 code: J40-J47)	present/absent
Chronic kidney diseases	Chronic kidney disease (CKD) (ICD-10 code: N18)	present/absent
Liver diseases	Diseases of liver (ICD-10 code: K70-K77)	present/absent
HIV infection	Human immunodeficiency virus [HIV] disease (ICD-10 code: B20)	present/absent
Dementia	Vascular dementia (F01),<br>Dementia in other diseases classified elsewhere (F02)	present/absent

© 2022 Wang W et al. *JAMA Oncology.*

	Unspecified dementia (F03)<br>Alzheimer's disease (G30)	
Substance use disorders	Mental and behavioral disorders due to psychoactive substance use (F10-F19)	present/absent
Depression	Depressive episode (F32)	present/absent
Anxiety	Anxiety, dissociative, stress-related, somatoform and other nonpsychotic mental disorders (ICD-10 code: F40-F48)	present/absent
Alcohol abuse	Alcohol abuse (F10.1)	present/absent
Tobacco use	Tobacco use (ICD-10 code: Z72.0)	present/absent
Stem cell transplant	Stem Cell Transplant (code 1005)	present/absent
Chemotherapy	Chemotherapy (code 1002)	present/absent
Targeted therapy	Targeted Therapy (code 1003)	present/absent
Radiation	Radiation (code 1001)	present/absent
Hormone therapy	Hormone Therapy (code 1004)	present/absent
Immunotherapy	Pembrolizumab (RxNorm code: 1547545), Nivolumab (1597876), Cemiplimab (2058826), Atezolizumab (1792776), Avelumab (1875534), Durvalumab (1919503), Ipilimumab (1094833)<br>Chimeric antigen receptor T-cell (CAR-T) therapy (CPT code 1035206)	present/absent
Pfizer-BioNTech vaccine	Immunization administration by intramuscular injection of severe acute respiratory syndrome coronavirus 2 (SARS-CoV-2) (Coronavirus disease [COVID-19]) vaccine, mRNA-LNP, spike protein, preservative free, 100 mcg/0.5mL dosage; second dose (CPT code: 0012A)	present/absent
Moderna vaccine	Immunization administration by intramuscular injection of severe acute respiratory syndrome coronavirus 2 (SARS-CoV-2) (Coronavirus disease [COVID-19]) vaccine, mRNA-LNP, spike protein, preservative free, 30 mcg/0.3mL dosage, diluent reconstituted; second dose (CPT code: 0002A)	present/absent
Johnson & Johnson vaccine	Immunization administration by intramuscular injection of severe acute respiratory syndrome coronavirus 2 (SARS-CoV-2) (coronavirus disease [COVID-19]) vaccine, DNA, spike protein, adenovirus type 26 (Ad26) vector, preservative free, 5x10^10 viral particles/0.5mL dosage, single dose (CPT code: 0031A)	present/absent

© 2022 Wang W et al. *JAMA Oncology.*

eTable 3: Characteristics of 45,253 vaccinated patients with cancer and 591,212 vaccinated patients without cancer. Shown were six most common cancer types in the database (breast cancer, prostate cancer, hematologic malignancy, colorectal cancer, skin cancer, lung cancer). Race and ethnicity were defined by TriNetX EHR database and included in the study since they are known to be associated with both risk and associated outcomes of SARS-CoV-2 infections. The status (present/absent) of comorbidities, SDOHs, and cancer treatments were based on anytime before to same day as index event of vaccination.

	**Breast cancer**	**Prostate cancer**	**Hematologic malignancy**	**Colorectal cancer**	**Skin cancer**	**Lung cancer**	**All Cancer**	**Not cancer**
**Total no. of patients**	13,032	11,421	6,962	3,094	2,926	2,849	45,253	591,212
**Age (Mean ± SD)**	68.3±11.7	72.4±8.85	65.9±15.6	70.1±12.3	68.1±13.5	71.4±10.7	68.7±12.4	51.1±20.9
**Gender (%)**								
Female	99.0	0.2	48.8	50.8	48.6	57.7	53.5	55.1
Male	1.0	99.8	51.2	49.2	51.4	42.3	46.5	44.9
**Ethnicity (%)**								
Hispanic/Latino	4.9	4.2	5.6	5.6	1.8	2.7	4.9	12.2
Not Hispanic/Latino	82.5	84.5	81.5	79.3	84.6	78.8	81.8	73.9
Unknown	12.7	11.3	12.8	15.2	13.6	18.5	13.3	13.8
**Race (%)**								
Asian	4.5	3.0	3.4	3.7	0.7	3.9	3.8	8.3
Black or African American	15.6	18.4	14.9	16.8	1.6	17.1	15.4	14.2
White	73.3	72.3	73.8	73.1	92.5	74.1	74.1	62.7
Unknown	6.2	5.9	7.5	5.9	5.1	4.7	6.3	13.8
**adverse social determinants of health (%)**	3.3	1.6	3.1	3.9	3.1	4.1	2.8	1.2
**Health conditions or behavioral factors (%)**								
Hypertension	50.9	59.2	54.4	59.8	56.6	65.0	55.8	17.9
Heart diseases	13.1	23.3	20.7	22.7	22.2	37.2	19.6	4.5
Cerebrovascular diseases	8.6	10.4	11.7	13.0	12.2	18.7	10.6	2.7
Obesity	21.4	16.0	19.3	22.2	21.3	20.2	20.6	8.0

© 2022 Wang W et al. *JAMA Oncology.*

Type 2 diabetes	17.6	20.7	21.1	25.1	17.6	24.3	20.9	6.6
Chronic respiratory diseases	20.0	15.8	23.3	22.1	20.2	53.0	21.0	6.7
Chronic kidney diseases	8.3	13.5	16.9	14.5	11.2	15.2	12.9	2.4
Liver diseases	12.3	9.9	15.7	20.4	14.7	21.1	14.5	1.9
HIV infection	0.1	0.7	1.9	1.1	0.7	0.9	0.8	0.3
Dementia	1.3	1.4	1.4	1.9	1.6	1.9	1.4	0.3
Substance use disorders	10.3	10.6	12.1	13.8	11.9	28.9	12.3	3.6
Depression	18.4	8.9	17.4	15.5	16.6	20.9	15.2	5.1
Anxiety	26.8	13.0	24.3	21.2	24.4	27.9	21.8	8.5
Alcohol abuse	3.8	3.2	2.9	3.7	3.9	5.5	3.6	0.8
Tobacco use	2.8	3.3	3.6	5.1	2.7	14.0	4.0	1.2
**Cancer treatment (%)**								
Stem cell transplant	0.3	0.4	10.5	0.4	0.7	0.4	1.7	NA
Chemotherapy	51.9	24.1	50.2	35.3	31.3	42.1	36.6	NA
Targeted therapy	44.0	6.3	35.1	14.4	18.2	26.5	23.3	NA
Radiation	28.3	18.0	11.5	13.2	9.2	27.2	17.7	NA
Hormone therapy	31.6	22.4	15.3	9.0	14.8	10.4	19.7	NA
Immunotherapy	0.4	0.5	0.7	1.0	5.7	8.9	1.4	NA
**Medical encounter for cancer within past year (%)**	58.0	61.3	60.6	46.6	38.3	62.8	58.5	NA
**Vaccine types (%)**								
Pfizer-BioNTech	81.6	80.2	80.8	80.6	76.3	78.9	80.9	75.3
Moderna	17.4	18.9	18.0	17.8	21.9	20.0	17.9	23.4
Johnson & Johnson	1.0	0.9	1.2	1.6	1.8	1.1	1.2	2.7

© 2022 Wang W et al. *JAMA Oncology.*

eTable 3 (continued): Characteristics of vaccinated population for thyroid cancer, bladder cancer, endometrial cancer, kidney cancer, liver cancer and pancreatic cancer. NA - not available. TriNetX does not report actual patient counts less than 10 for security reasons.

	**Thyroid cancer**	**Bladder cancer**	**Endometrial cancer**	**Kidney cancer**	**Liver cancer**	**Pancreatic cancer**
**Total no. of patients**	2,329	2,272	1,698	2,093	877	627
**Age (Mean ± SD)**	60.9±14.9	74.1±10.0	68.4±10.4	68.1±12.5	66.5±11	70.2±10.9
**Gender (%)**						
Female	73.9	28.1	100.0	41.4	38.3	48.6
Male	26.1	71.9	NA	58.6	61.7	51.4
**Ethnicity (%)**						
Hispanic/Latino	6.6	3.3	5.1	7.1	9.2	4.1
Not Hispanic/Latino	78.8	83.3	76.7	80.4	67.3	82.1
Unknown	14.6	13.4	18.2	12.5	23.5	13.7
**Race (%)**						
Asian	6.5	2.8	3.4	2.5	8.2	4.0
Black or African American	12.1	9.3	17.3	16.8	16.9	17.9
White	72.1	82.7	73.9	73.8	67.3	70.0
Unknown	8.9	5.0	5.1	6.4	7.4	7.5
**adverse social determinants of health (%)**	2.9	2.2	3.7	3.0	5.7	2.6
**Health conditions or behavioral factors (%)**						
Hypertension	50.3	67.1	57.1	71.1	69.9	64.1
Heart diseases	13.7	33.9	13.6	29.1	29.9	26.0
Cerebrovascular diseases	9.0	13.8	7.4	13.9	14.8	13.1
Obesity	26.4	20.7	41.2	29.0	28.2	21.2
Type 2 diabetes	20.7	26.6	26.3	30.1	30.1	36.2
Chronic respiratory diseases	21.1	27.7	21.1	25.0	29.8	25.2
Chronic kidney diseases	9.4	22.2	10.4	34.7	28.5	16.3
Liver diseases	12.9	19.3	15.7	30.0	74.6	34.8
HIV infection	0.4	0.8	NA	0.9	2.3	NA
Dementia	0.9	2.1	1.4	1.4	NA	NA
Substance use disorders	9.6	18.2	7.8	16.8	30.3	16.4
Depression	18.2	15.1	18.7	18.3	20.0	19.1

© 2022 Wang W et al. *JAMA Oncology.*

Anxiety	26.7	19.3	24.2	23.7	26.7	24.6
Alcohol abuse	1.9	4.0	1.5	0.5	2.1	NA
Tobacco use	2.2	5.9	2.5	5.1	10.1	4.9
**Cancer treatment (%)**						
Stem cell transplant	0.4	NA	NA	NA	NA	NA
Chemotherapy	18.4	41.7	31.7	27.3	43.6	47.8
Targeted therapy	11.9	16.6	15.1	19.0	23.6	17.1
Radiation	14.6	7.9	24.0	9.4	16.2	14.5
Hormone therapy	25.3	12.1	11.2	11.8	9.6	10.4
Immunotherapy	0.5	3.3	1.2	6.8	3.9	1.9
**Medical encounter for cancer within past year (%)**	54.6	57.1	45.2	50.7	50.4	58.2
**Vaccine types (%)**						
Pfizer-BioNTech	83.9	78.3	80.9	80.4	73.2	75.3
Moderna	14.2	20.9	17.4	18.8	25.5	24.4
Johnson & Johnson	1.8	0.8	1.7	0.8	1.3	NA

© 2022 Wang W et al. *JAMA Oncology.*

eFigure: Hazard ratios for breakthrough SARS-CoV-2 infections in vaccinated patients with cancer who had recent medical encounters for their cancer diagnosis within the past year between November 2020-November 2021 ("Cohort-1") as compared to propensity-score matched vaccinated patients with cancer who had no medical encounters for their cancer diagnosis within the past year ("Cohort-2"). Breakthrough SARS-CoV-2 infections were followed in both cohorts starting14 days after full vaccination up to November 30, 2021. Two cohorts were matched for demographics (age, gender, race/ethnicity), SDOHs, comorbidities, vaccine types and cancer treatments.

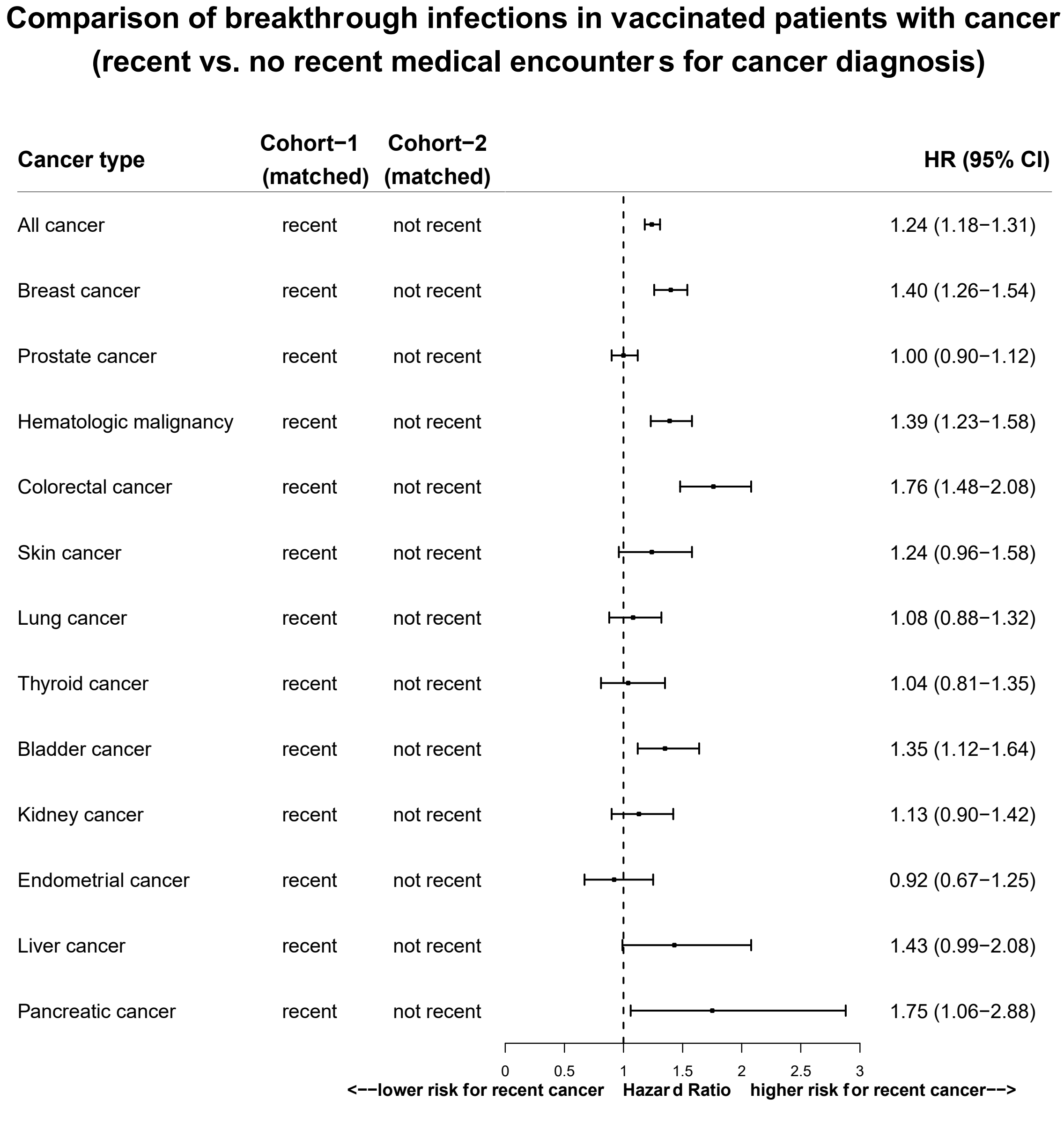

© 2022 Wang W et al. *JAMA Oncology.*
